# Creating Digital Health Technology for Gender Equity: a Capabilities Approach

**DOI:** 10.1093/phe/phaf012

**Published:** 2025-08-22

**Authors:** Naomi Jacobs

**Affiliations:** University of Twente, Enschede, the Netherlands

**Keywords:** Gender equity, women’s health gap, digital health, capabilities approach, capability sensitive design

## Abstract

This article examines the role digital health technology plays in the perpetuation of gender inequity in health and presents a capability-sensitive design approach to actively promote health equity in digital health technology design. The article shows how a capability-sensitive design approach, with its central focus on human diversity and conversion factors, can assist health technology designers in creating digital health that serves people equally and thereby fosters equity and social justice in health.

## Introduction

Health inequities between women^1^ and men persist on a global scale to this day (World Economic Forum, 2024). A recent research report on the Women’s Health Gap by the World Economic Forum (WEF) (2024) demonstrates that women spend 25 per cent more of their lives in poor health in comparison to men, despite living longer than men. An androcentric perspective has long dominated medical research, taking the male body as the ‘human standard’ from which medical findings, therapies and medicine are generalized (see e.g. Criado Perez, 2019; Dusenbery, 2019; Gleghorn, 2021). For example, most animal models used for medical research have been based on male animals (Zucker and Beery, 2010), and medicine has worked long with the assumption that there are few important differences in the functioning of organs and systems in men and women beyond reproduction (World Economic Forum, 2024). We now know this assumption to be false: research increasingly shows that women differ biologically, as well as physically and behaviorally from men (Mauvais-Jarvis et. al., 2020). Women and men, for instance, can experience important differences in the uptake or effectiveness of a medicine designed and approved for use for both. This is the case for certain therapies to treat asthma and cardiovascular disease. Researchers of the WEF report on the Women’s Health Gap examined:

‘183 of the most widely used interventions across 64 health conditions, representing roughly 90 per cent of the health burden for women, reviewing more than 650 academic papers to assess the extent of this phenomenon. Of the interventions studied, only 50 per cent reported sex-disaggregated data. In cases where sex-disaggregated data were available, 64 per cent of the interventions studied were found to put women at a disadvantage, either due to lower efficacy or access, or both, while for men this was the case for only 10 per cent of interventions’ (World Economic Forum, 2024).

In response to these alarming facts on women’s health, researchers and policymakers have been considering solutions to solve the problem of the women’s health gap. Solutions that are proposed in the WEF’s Women’s Health Gap report include integrating sex and gender differences into research, e.g. by identifying sex and gender differences in the mechanism, disease or treatment under study, and by identifying methods for integrating sex and gender variables in health research contexts (Canadian Institutes of Health Research, 2019). As well as stimulating women in leadership positions with performance and impact indicators; strengthening the representation of women in clinical trials in tune with disease prevalence; establishing new types of research and development partnerships that can lead to new innovation and medical solutions to address major unmet needs in women’s health, such as endometriosis; and employee policies and benefits that promote women’s health and cultivate environments where women feel empowered to speak openly about their health needs (World Economic Forum, 2024).

Although these are extremely valuable solutions offered to solve the problem of the women’s health gap, these solutions do appear to overlook a crucial factor at play in the problem. That is: these solutions largely overlook the role that *digital health technology* plays in the perpetuation—and exacerbation—of the women’s health gap. Digital health technology, often abbreviated as *digital health*, concerns digital technologies that deliver some sort of healthcare services with the aim to improve health. Digital health, however, is rarely designed with a genuine focus on fairness in the distribution of health advantages and the different needs of genders (Crawford and Serhal, 2020). Consider the following illustrative example of a gender-biased algorithm implemented into the digital health app ‘Babylon’ that was released by the UK National Health Service in 2017. The Babylon app is a digital health app for medical decision making and consists of a chatbot providing a ‘general practitioner at hand’ service and remote chatbot consultations (Trendall, 2019). The digital health app works with artificial intelligence to advice patients on the probability of a diagnosis based on their self-reported symptoms and subsequently advises a course of action: contact a doctor, visit the emergency room or no action required (Figueroa et al., 2021). Now, an experiment with the Babylon app widely shared on social media revealed that the app provided markedly different diagnostic possibilities for a female and a male patient who had the same background and symptoms. That is: a *female* smoker aged 59 years with symptoms of a heart attack (i.e. chest pain, shortness of breath and anxiety) was provided with a diagnosis of depression or panic attack, whereas a male smoker with the same background and symptoms was informed of the possibility of a heart attack (Trendall 2019; cited in Figueroa et al., 2021). What this example illustrates is that the symptoms of the female smoker are misinterpreted or taken less seriously than those of the male smoker, with the possibility of the female smoker receiving an incorrect diagnosis in a life-threatening situation. Women still rarely receive the same benefits from digital health as men do. This is for a part caused by the fact that the specific influence of digital technology design on health outcomes and health inequities has, as of yet, received little attention. In a recent publication, Chidambaram et al. state that ‘there is a paucity of literature addressing how the intrinsic design, implementation and use of technology interact with social determinants of health to influence health outcomes’ (Chidambaram et al., 2024: 1).

In this article, I argue that it is crucial to examine the role of digital health technology design in the continuation—and exacerbation—of the women’s health gap. Medical research and healthcare increasingly rely on digital health. However, the ways in which digital technologies are designed and implemented is never value neutral. Instead, technologies reflect assumptions, views and values via ways in which these technologies are designed and function. Critical reflection is needed upon the questions *who* is designing *what* kind of technology, and for *whom* a technology is primarily intended and for whom not. To fully understand the current women’s health gap and its effects on inequity between women and men in health, we need to explicitly examine the role of digital health technology design. Moreover, this article will explore a capabilities approach to provide us with a clearer understanding of (in)equity in relation to health and digital health technology design in particular. A capabilities approach takes capabilities as a metric of equity. A capability refers to a valuable opportunity or positive freedom available to people. According to philosopher Martha Nussbaum, there are ten basic capabilities in life that every person should have access to in order to life a worthy and dignified life (Nussbaum, 2011; Jacobs, 2020). Several of these basic capabilities concern good health. However, as the WEF report (2024) shows, women on average experience fewer advantages from medical research and healthcare than they ought to enjoy given how other members of society (i.e. men) are faring. The basic capabilities of health are thus less easily available for women. This unequal access to and optimal experience of the capability of health should be understood, as will be argued in this article, as an injustice.

Please note that in this paper, the focus is on the women’s health gap and gender inequity; however, the intent is that the focus on gender inequity functions as a discussion starter on intersectional inequities and the challenge of addressing inequities in health technology design to eliminate health disparities and health gaps in general.

## Equity in Health

Equity in health is widely defined as the absence of socially unjust or unfair health disparities (Whitehead, 1992). More specifically, equity in health refers to the absence of systematic unjust disparities in health between individuals or social groups who have different levels of underlying social advantage or disadvantage (Braveman and Gruskin, 2003). Health inequities systematically put populations, social groups or individuals who are already disadvantaged at further disadvantage with respect to their health (Braveman and Gruskin, 2003). Important to note is that there is a difference between health *inequity* and health *inequality*; the latter refers to observable differences in health among individuals or groups. However, not all differences in health are necessarily unjust. For example, we may expect on average that young adults are healthier than elderly people, or men to have prostate problems while women do not (Braveman and Gruskin, 2003). Such differences in health between individuals or groups of people are observable, but not unfair. Health inequities, on the other hand, always refer to socially unjust health disparities. For instance, differences in the likelihood of receiving appropriate treatment for cardiovascular diseases between women and men, or differences in the likelihood of receiving a right and timely diagnosis for skin cancer between people with darker and fair skin, are examples of health differences that are socially unjust from an equity perspective (Braveman and Gruskin, 2003). As argued by health equity scholars Paula Braveman and Sofia Gruskin:

‘Equity in health means equal opportunity to be healthy, for all population groups. Equity in health thus implies that resources are distributed, and processes are designed in ways most likely to move toward equalizing the health outcomes of disadvantaged social groups with the outcomes of their more advantaged counterparts’ (Braveman and Gruskin, 2003: 257).

Concerning the women’s health gap, we can state that *gender* inequity in health directs our attention to the fewer health advantages that women enjoy, as well as more of the health burdens they bear than they ought to bear in comparison to how men are faring in society. The distribution of advantages and disadvantages with respect to health between men and women is, as shown by the recent WEF’s report on the women’s health gap, systematically unjust (World Economic Forum, 2024). As indicated by the report, the systematically unjust distribution of (dis)advantages with respect to health is heavily influenced by the social determinants of health. These are the non-medical determinants that influence and effect health outcomes and health inequities. Widely acknowledged is that these social determinants of health include, e.g. the conditions in which people are born, grow, work, live and age, and the wider set of forces and systems shaping the conditions of daily life. These forces and systems include economic policies and systems, development agendas, social norms, social policies and political systems (WHO, 2024). The specific influence of digital technology design on health outcomes and health inequities has, yet, received few attention (Chidambaram et. al. 2024: 1). This statement can, however, be nuanced somewhat as a recent scoping review by Petretto et al. (2024) identifies eight papers that investigate the relationship between so-called ‘digital determinants of health’ and social determinants of health. Richardson et al. (2022), for instance, put forward a framework for digital health equity. The authors speak of digital determinants of health as to include access to technological tools, digital literacy and community infrastructure like broadband internet and emphasize that these digital determinants of health ‘likely function independently as barriers to and facilitators of health as well as interact with the social determinants of health (SDoH) to impact outcomes’ (Richardson et al., 2022: 1). Now, what still receives little attention, however, is attention to how the *design* of digital health technology can be a digital determinant of health as well. That is: the design of digital health—e.g. how a technology functions, what features it bears, how it gathers, processes and presents data—often functions as a barrier or facilitator of health. A rich body of scholarly literature in philosophy of technology has argued that technologies are never value neutral (see e.g. Latour 1993, 1994, 2005; Akrich 1992), instead, philosophers of technology such as Bruno Latour argue that technologies can authorize, allow, afford, encourage, permit, suggest, influence, block, render possible, forbid and so on human action (Latour, 2005). This indicates that human actions result not only from individual intentions and the social structures in which we find ourselves but also from *technologies themselves* (Latour, 2005). Technologies—to certain extents—always reflect social views, moral assumptions and values in their functionalities and design implications. As argued by Van den Hoven et al., the many decisions that designers make during a design process affect not only the technology’s functionality, usability and aesthetics, but also shape and affect the interactions and constraints of a technology to its users Van den Hoven et al. (2015). Therefore, we should start to understand technology design as a digital determinant of health. We need to examine how the designs of digital health technology can function as barriers to and facilitators of health and how design interacts with other digital and social determinants of health, influencing health outcomes and health equity. We need to explicitly reflect upon the question who is designing what kind of technology, and for whom a technology is primarily intended and for whom not. In relation to health inequity and the women’s health gap, it is of crucial importance that we start to carefully examine how digital health technology design effects health outcomes and health inequities.

## Health Technology Design and Gender (In)equity

Digital health technology concerns digital technologies that deliver some sort of healthcare services with the aim to improve health. More specifically, digital health can be understood to refer to technologies that engage people with health and wellness purposes, collect and use their (clinical) data and manage health outcomes and quality of care (Chidambaram et al., 2024). Artificial intelligence for health, personalized medicine, mobile health and health information systems, amongst others, are examples of digital health.

Various scholars have emphasized the empowering potential that digital health can have, especially for women. It has been argued, e.g., that digital health has the potential to address gender inequity in health by ensuring increased access to healthcare, sharing of health information and services, empowerment of one’s health data and reduced burden of unpaid care work (Figueroa et al., 2021). Over the past years, there has been a large increase in health apps specifically targeted at women to track their menstrual cycle and fertility. These so-called *femtech* (short for female technology) apps are said to have the potential to bridge the current gender data gap. Women who make use of these menstrual apps—e.g. the app *Clue*—contribute to a large data set on female health that the creators of the app, together with various research institutes and clinicians, use to come to a better understanding of female bodies (Shea, 2025). Furthermore, various authors have pointed out that these digital health apps provide women with information and insights into their menstrual cycle and fertility and thereby allow them to become more informed about their own health and help them take charge over their own bodies and health outcomes (Jacobs and Evers, 2023).

However, there are just as many scholars who have pointed out that digital health is rarely designed with a genuine focus on fairness in the distribution of health advantages and the different needs of genders (Crawford and Serhal, 2020). Moreover, as is argued, digital health often treats women unfairly, especially those with minority backgrounds (Figueroa et al., 2021). For example, in the creation of digital health, research questions concerning minority or disadvantaged groups are often not prioritized, which may cause structural biases to translate into the digital health design. Chidambaram et al. point out this may happen because the dataset that a digital health technology works on is itself underrepresented; or it is developed based on representative data but applied to the unintended minority population; or the data used may not account for social categories and determinants of the intended outcome; or the data used may have sociohistorical bias in terms of how it was entered and collected (Chidambaram et al., 2024). These types of bias can all lead to digital health outputs that treat those who fall outside of the ‘standard mold’ with much less accuracy, leading to poorer health outcomes for people with minority backgrounds who are often already affected by health disparities (Chidambaram et al., 2024).

In what follows, an example of a digital system for medical diagnosis is presented to show how a digital health technology can perpetuate—and even exacerbate—existing gender inequities in health through its design.

Imagine you are Dewi, a woman in her mid-thirties with Indonesian roots and mother of two young boys. You have been rather tired for some time now, you have a fever every now and then and recently you have developed red spots on your face and neck. You decide to go to your GP, who listens to you and enters your symptoms into a digital system for medical diagnosis that your GP uses as assistance for making medical diagnoses. The diagnosis that results from the digital system is severe flu. The advice that your GP gives you based on this result is to take some extra vitamin C and enough rest. This diagnosis, however, does not correspond with your own lived experience, but the doctor assures you that your symptoms have just been compared in the digital system with a large amount of data on medical advice for similar symptoms. Based on this large dataset, the medical diagnosis of influenza is established, and your GP tells you to trust it. What’s really going on, however, is that Dewi suffers from the autoimmune disease systemic lupus erythematosus. It is known that this disease can cause relatively general and wide-ranging symptoms, and it usually takes several years before the correct diagnosis is made. The cause of the disease is currently still unknown. What we do know is that the disease occurs mostly in women, particularly in Asian and black women.

Let us take a closer look at the specific role that the digital health system for medical diagnosis plays in this example. A GP with a lack of knowledge on a women-specific disease, such as systemic lupus erythematosus, would perhaps *without*, the assistance of a digital system, make one or more wrongful diagnoses for their patients. A digital system that works with an incomplete or asymmetrical dataset can easily make over a hundred or even a thousand wrongful diagnoses in similar cases. When doctors increasingly rely on digital systems that are based on incomplete knowledge and asymmetric datasets, existing inequities are quickly amplified on a much larger scale. Not surprisingly, asymmetrical datasets are common in the health domain as there is more knowledge and data available about what has been understood to be ‘the human standard’ for centuries on end, i.e. the white middle-aged male. Asymmetrical datasets show a health data poverty in knowledge about marginalized groups, including women’s health. Several studies have addressed this problem of incomplete and asymmetrical datasets in digital health technologies and examples include: a study aimed at predicting acute kidney injury, the used data model severely underperformed in female patients as only 6.4 per cent of its initial dataset were from female patients (Tomas et al. 2019); symptom checkers, which are built on large datasets, but these are usually not published for scrutiny and so may not necessarily incorporate minority groups (Knitza et al., 2021); similar instances of underrepresentation have been seen in diagnosing skin lesions, as most algorithms do not include skin lesions in ethnic minorities (Du-Harpur et al., 2020; cited in Chidambaram et al. 2024). Instead of contributing to the closing of the women’s health gap, such digital technologies perpetuate and even exacerbate the digital divide through health data poverty and asymmetry.

Digital health, such as the above-discussed example of the digital system for medical diagnosis, furthermore raises the problem of explainability. Many digital technologies apply algorithms that are trained on large datasets, which are often not only incomplete or asymmetrical, as just discussed but are also increasingly difficult to explain as the calculations that are carried out by these algorithms are often ‘not assigned an easily understandable meaning, aside from there being far too many of these calculations to actually follow. The outputs of algorithms are, as a result, hard to predict and to explain’ (Buijsman, 2023: 1). For one thing, this raises the question of how someone could question or object to a diagnosis if it does not correspond with their lived experience, as is the case in the example of Dewi. When your GP makes a diagnosis, it is possible to have a conversation and ask your GP how a certain conclusion is made—and if necessary, request a second opinion. When a complex algorithm makes a diagnosis, it is much more difficult—if not impossible—to have a genuine conversation about a diagnosis, let alone question or even object to a given diagnosis. In addition, there is the problem of automation bias. Research has shown that people often defer to automated systems, thereby reducing the amount of independent scrutiny that they exhibit when making decisions. Due to automation bias, people often either follow a system’s advice even though it is incorrect and there is contradicting evidence, or, instead, people deviate from a system’s advice based on strong beliefs that suggest otherwise, even though the system is actually right (Green, 2022). In Dewi’s case, the GP follows the diagnosis provided by the medical system, thereby adhering to the results of the system even though Dewi’s testimony of her own lived experience contradicts the diagnosis. As a result of such automation bias, whereby a GP hardly deviates from the system’s results, it becomes even more difficult for Dewi to have a genuine conversation about her diagnosis, let alone question or object to it.

Wrongful diagnoses, due to discriminatory ideas, false beliefs or incomplete and asymmetrical data sets upon which our digital health systems rely, are made on a much larger scale due to the vast scale of applicability of these digital systems, their black-box character and automation bias. The ways in which digital technologies are designed and implemented are never value neutral. Instead, digital systems such as the one discussed in Dewi’s example above are designed on the incorrect belief that the male body can function as a human standard from which diagnoses for all people can be derived. To address (gender) inequity in health, we need to explicitly question the often-hidden assumptions that lay at the core of technology designs. We should critically reflect upon *who* is designing *what* kind of technology, and for *whom* that technology is primarily intended and for whom not. In the next section, a capability-sensitive design (CSD) approach to health technology design is discussed, as to provide a systematic approach to proactively stimulate the value of (gender) equity in the design of health technology.

## A Capabilities Approach to Health Technology Design

In recent years, it has been increasingly recognized that designers and engineers can actively represent, support or undermine values in their technology designs (see e.g. Brey, 2012; Van den Hoven et al., 2015; Friedman and Hendry, 2019). In response to this growing awareness, various anticipatory ethics and ethics-by-design approaches have emerged that aim to provide practical guidance to designers and engineers on how to proactively design for values and ethical considerations in their technology designs (see e.g. Brey, 2012; Friedman et al. 2013; Wynsberghe, 2013; Friedman and Hendry, 2019; Cenci and Cawthorne, 2020; Hoepman, 2018; Jacobs, 2020). What these approaches have in common—despite their diversity—is their endorsement of the fact that design decisions shape and affect the set of interactions and constraints of a technology to users, and these decisions can support or undermine (moral) values. In other words, values can be expressed and embedded in technology through design choices made (Van den Hoven et al., 2015). What these approaches furthermore share is the idea that conscious and explicit thinking about the values that are imparted to our technological inventions is morally significant and that such considerations should be articulated proactively and early in the design process; at the moment of the design and development when they can still make a difference (Van den Hoven et al., 2015).

CSD is one such ethics-by-design approach that deserves our attention, as it is a promising approach to the problem of (gender) inequity in the context of health technology design (Jacobs, 2020). CSD is a capabilities approach to the design of health technologies that explicitly focuses on fostering the moral values of social justice and equity in technology design. The approach combines the methodology of value sensitive design (Friedman and Hendry, 2019) with the moral foundations of Martha Nussbaum’s capability theory of social justice (Nussbaum, 2011). Nussbaum’s capability theory of social justice is built upon the two premises that (i) all people are morally equal and deserve a life worth living, and (ii) that everyone is entitled access to 10 basic capabilities in order to live such a worthy life. In contrast to, e.g. social contract theories that adopt social primary goods such as opportunities, basic liberties and income as their metric for assessing an individual’s comparative advantage over others in society, the capabilities approach focuses on what people are actually able to be and do—their capabilities and functionings—as metric for social justice and equity (Putnam et al., 2019).

Feminist philosophers and critical disability scholars have firmly criticized social contract theories or egalitarian resource-based approaches to distributive justice for not considering the environment in which resources and goods are to be utilized, while this profoundly affects the value of those goods to the people receiving them. As two persons might be alike in their share of social primary goods, and have similar projects, aims, commitments and values, but nonetheless differ in the value they can derive from those goods, if one person is disabled and the other is not (Putnam et al., 2019). The capabilities approach is not concerned with the number of goods or resources an individual has available but instead focuses on what someone *can do* with those resources as a metric for social justice, thereby carefully taking into account the role of the environment and people’s diverse personal abilities to convert resources into capabilities and functionings. At the core of the capabilities approach lies the insight that resources not necessarily lead to increased capabilities, but instead, personal, social and environmental *conversion factors* greatly influence the degree to which a person can transform a resource into capabilities and realized capabilities, called ‘functionings’. With the term ‘conversion factors’ the capabilities approach refers to those personal, social and environmental factors that determine the degree to which a person can transform a resource into a (realized) capability (Robeyns, 2017). There are noteworthy resemblances between the concept of conversion factors and social determinants of health. Whereas social determinants of health refer to all the non-medical factors that influence health outcomes, conversion factors refer to an individual’s personal, social or environmental ability to convert a resource—such as a digital health technology—into the expansion of a capability, such as the capability of health. Moreover, with the emphasis on the importance of considering people’s personal, social and environmental conversion factors and their interrelation, CSD is able to offer an intersectional perspective on the design of digital health. Intersectionality refers to the notion that structures of disadvantage do not exist independent of one another but are constituted by multiple and intersecting relationships of power associated with people’s race, sex, gender, class, caste, religion, sexual orientation, disability, age, marital status etc’ (Crenshaw, 1989). By putting conversion factors central in the design perspective, CSD gives explicit notice to the idea that structures of disadvantage do not exist independent of each other but are an interplay of people’s personal, social and environmental conversion factors.

Nussbaum has identified ten basic capabilities that she defends as being the moral entitlements of every human being to live a worthy and dignified life^2^ and several of these capabilities have to do with health, such as the capabilities of being able to live a normal length of lifespan; having good health; maintain bodily integrity; and being able to use the senses, imagination and think (Nussbaum, 2011). Nussbaum’s capability theory of social justice argues that every human is equally entitled access to these ten basic capabilities. However, systematic unjust health disparities between men and women, and among women, persist. From a capability perspective, this unequal access to and optimal experience of the capabilities of health is clearly to be understood as a social injustice. When the capability to live in good health is identified as a necessary capability for a worthy and dignified life for all people equally, then everyone should be enabled access to and an optimal experience of that capability. Thereby, carefully taking personal, social and environmental conversion factors into account, as individuals have different abilities to convert resources into capabilities, which plays a crucial role in access to and experience of the capability.

A CSD approach to the design process of health technology starts with explicit reflection on the following set of questions: what capability—or capabilities—are to be expanded with the new technology? For whom is the new technology primarily intended, and what capabilities do these people value? Answering these questions requires conceptual analysis, but most importantly, empirical stakeholder engagement. Through empirical inquiry, designers are to examine what capabilities are valued by their intended user group, as well as how these capabilities are understood and specified in relation to the lived experiences of these people. In addition, designers should empirically investigate the diverse abilities of the people in their intended user group, to get a clear view of the personal, social and environmental conversion factors that play a role for these people in converting the new technology into actual expansion and enhancement of their capabilities. The (implicit) assumption that there is one human default that can be used as a vantage point in health technology design—an assumption persistently present in medicine and technology design—is quickly disproved by empirical investigation into people’s diverse conversion factors. Important to note here is that feminist STS scholars have argued that researchers and designers should always take a reflexive approach to empirical, participatory design practices (Lysen and Wyatt, 2024). This entails critical reflection on how designers are themselves constituted in the design project; the ways in which they reach out to and select participants; the ways in which they are not only teaching but also learning from the participants involved; and what power, resource or other asymmetries may characterize the contexts within designers seek to intervene (Conley and York, 2020). A CSD approach asks from practitioners to take on such a reflexive approach to their empirical participatory methods, and to the design process as a whole. In addition to such reflexive empirical inquiry, designers engage in technical investigation through prototyping based on their empirical and conceptual findings. Technical adjustments can be made to initial designs, based on the empirical findings on conversion factors of the people in their intended user group. These technical prototypes should then be empirically tested with the intended user group and, through an iterative process, be readjusted accordingly.^3^

By taking on the CSD approach, designers can signal any moral injustice in the technology design at hand when it becomes apparent during the design process that the technology insufficiently serves a subgroup of the intended user group, either because relevant conversion factors of this subgroup are not adequately taken into consideration or because certain conversion factors of this subgroup are not adequately translated into technical requirements of the design. This directs us to a moral injustice, as at the start of the design process, one or more capabilities are selected to have moral value in the design context at hand and to be made accessible to everyone in the intended user group. The design process must therefore facilitate all people in the intended user group to have equal access to these selected capabilities through the technology design.

A well-known example of technology design that has long *failed* to acknowledge such moral injustice is the design of cars. The standard seating position for which cars long have been designed is based on the average men’s anatomy. Even though women are, on average, shorter than men. Women, therefore, need to sit closer to the steering wheel to reach the pedals and more upright to see clearly over the dashboard, which places women at a greater risk of injury when involved in a car crash in comparison to men (Criado Perez, 2019). In theory, cars are designed for a large user group, including men *as well as* women. In practice, car designs have long failed to sufficiently consider the relevant conversion factors of all the people in that user group, namely the personal conversion factors of most women, having to do with the fact that women’s bodies are, on average, shorter than men’s. The result is that women are at an increased risk of injury when making use of the design. Paying close attention to the relevant conversion factors of *all* people in the intended user group helps designers to adequately account for human diversity and avoid moral injustice to be instilled in their designs (Jacobs, 2020). What the CSD approach thus enables designers to do is: (i) signal moral injustices in a technology design during the design process, and (ii) proactively take human diversity into account during the design process by focusing on conversion factors, thereby creating health technology that serves people fairly.

Let us once more look at the example of the digital system for medical diagnosis discussed in the Health Technology Design and Gender (In)equity section, but now from a CSD perspective. What differences is a capability approach to the design of such a digital health system for medical diagnosis able to make? Taking on a capability perspective requires from designers to start their design process with asking not only the question what kind of technology to design, but also immediately focus on the questions what capabilities this technology aims to expand, and for whom?^4^

The digital system for medical diagnosis is a technological system that aims to assist GPs with medical diagnoses. We may assume here that the technology aims to enhance the capabilities of being able to live a normal length of lifespan and of having good health. Everyone visits a GP at some point in their life, the technology thus applies to a very extensive user group including people from all ages, all genders, all socio-economic backgrounds, ethnicity or educational background, to name just a few relevant social positions people from the intended user group inhabit.

A CSD perspective stimulates designers to carefully consider the personal, social and environmental conversion factors of the people for whom a technology design is intended. In case of the digital system for medical diagnosis, the social conversion factors of digital literacy and educational background^5^ play a role in how well someone can understand the workings of a digital system and someone’s ability to converse about the information and diagnoses provided by the system with their GP. Someone with high digital literacy, a high educational background and strong communication skills will most likely be able to better communicate with their GP about the diagnosis they received from the digital system when it does not correspond with their own lived experience (and possibly object and request a second opinion), than someone who has little digital literacy and educational background to take on such a conversation. Social conversion factors of digital literacy and educational background thus influence the degree in which someone is able to convert the digital system for medical diagnoses into the expansion of the capabilities of living a life of normal lifespan and having good health, and designers of such a system should carefully take this into account as people from the intended user group possess different (degrees of these) social conversion factors. From a CSD perspective, design solutions are to encourage and facilitate a good conversation between GP and patient about the diagnosis provided by the digital system. To account for the social conversion factors of digital literacy and educational background, design solutions could, e.g. include an additional chatbot function for patients that facilitates an accessible conversation about the diagnosis and helps the patient to formulate questions and thoughts that they can use to start a conversation with their GP. Also, it could include the design of visual tools that support the explanation of the diagnosis and serve as a conversation starter between GP and patient.

A CSD approach thus prescribes that designers place careful analysis of people’s conversion factors and their interconnectedness at the heart of their design process, as CSD makes apparent that people have diverse abilities to convert resources into a capability. Moreover, with this strong emphasis on the importance of people’s diverse conversion factors and their interrelation, CSD offers designers an intersectional perspective on how structures of (dis)advantage do not exist independently of one another but are constituted by multiple and intersecting relationships of power associated with people’s diverse conversion factors. Here, it is the social conversion factors of digital literacy and educational background that together influence the degree to which a person can convert the digital system for medical diagnoses into the expansion of capabilities of health. The primary focus of this paper has been on gender inequity in digital health, but as the above indicates, CSD is able to offer a much-needed perspective on digital health that identifies intersectional health issues and accounts for them in the design of these technologies.

## Concluding Remarks

This article focused on the role of digital health in perpetuating the women’s health gap and has put forward a CSD approach to digital health to mitigate health inequity and actively promote health equity. The primary focus of this article has been on women’s health; however, ‘women’ are not one homogeneous group just as men are not. Women differ significantly in bodily and mental abilities as well as in social positions and environmental placements. A capabilities approach emphasizes the importance of thinking in terms of people’s personal, social and environmental conversion factors, which explicates people’s diverse abilities and positions. Every individual has a unique profile of personal, social and environmental conversion factors, which enables him or her in a unique way to convert any given good—such as a digital health technology—into a capability or functioning. A capability perspective on digital health design offers an intersectional perspective in digital health design and emphasizes the importance of taking human diversity into account, because only then can technology be created that serves people’s diverse health needs fairly. The article has proposed to understand digital health design as a digital determinant of health that interacts with other digital and social determinants of health, affecting people’s health outcomes. The article aimed to convince the reader that the complex interactions between design and other digital and social determinants of health is an important topic for future study. Furthermore, the article aimed to convince the reader that a CSD approach with its central focus on conversion factors can move designers away from creating digital health technologies that primarily serve the white male ‘default’ body, and instead help them design digital health that serves people more equally and foster equity and social justice in health.

## Footnotes

1 Throughout this paper, the term ‘women’ is used to refer to people with female biology traits as well as people who identify as women.

2 In the capability approach literature, there is ongoing debate about whether the approach should have a list of capabilities, as Nussbaum proposes, or not. This article endorses Nussbaum’s capability theory and the capability-sensitive design framework as put forward by Jacobs (2020). It is beyond the scope of this paper to discuss the debate in depth here. For more on this debate, please see e.g. Robeyns (2017).

3 It is beyond the scope of this paper to discuss the various phases of the capability-sensitive design process in detail here for a more detailed account, please see (Jacobs, 2020).

4 Please note that this is a very brief retrospective application of the capability-sensitive design approach to the digital system for medical diagnosis. A comprehensive application of the capability-sensitive design approach takes place proactively at the start of a new design process and contains conceptual analysis as well as empirical and technical investigations in an iterative and mutually informing process as described above. Through reflexive empirical inquiry with stakeholders, designers investigate what capabilities are morally relevant for the design context at hand, how stakeholders specify these capabilities and what conversion factors are at play for these stakeholders.

5 As mentioned earlier on page 6, these social conversion factors closely resemble the social and digital determinants of health of (digital) literacy and educational background.
