# Correction to: Creating Digital Health Technology for Gender Equity: a Capabilities Approach

**DOI:** 10.1093/phe/phaf018

**Published:** 2025-10-08

This is a correction to: Naomi Jacobs, Creating Digital Health Technology for Gender Equity: a Capabilities Approach, *Public Health Ethics*, Volume 18, Issue 3, November 2025, https://doi.org/10.1093/phe/phaf012

The following changes have been made to the originally published manuscript.

The first footnote is moved in position to be after “women” within the first sentence of section **Introduction**.

In section **A Capabilities Approach to Health Technology Design**, text in the first sentence of the eighth paragraph: “Let us once more look at the example of the digital system for medical diagnosis discussed in the Equity in Health section, but now from a CSD perspective.” now reads instead: “Let us once more look at the example of the digital system for medical diagnosis discussed in the Health Technology Design and Gender (In)equity section, but now from a CSD perspective.”

Footnote 5 is emended to read: “As mentioned earlier on page 6, these social conversion factors closely resemble the social and digital determinants of health of (digital) literacy and educational background.” instead of: “As mentioned in the Health Technology Design and Gender (In)equity section, pages 10–11, these social conversion factors closely resemble the social and digital determinants of health of (digital) literacy and educational background.”

The emendations have been made to the article.

